# Taming a wandering attention: short-form mindfulness training in student cohorts

**DOI:** 10.3389/fnhum.2013.00897

**Published:** 2014-01-06

**Authors:** Alexandra B. Morrison, Merissa Goolsarran, Scott L. Rogers, Amishi P. Jha

**Affiliations:** ^1^Department of Psychology, University of MiamiMiami, FL, USA; ^2^School of Law, University of MiamiMiami, FL, USA; ^3^Mindfulness Research and Practice Initiative, University of MiamiMiami, FL, USA

**Keywords:** mindfulness training, mind wandering, working memory training, cognitive training, sustained attention

## Abstract

Mindfulness training (MT) is a form of mental training in which individuals engage in exercises to cultivate an attentive, present centered, and non-reactive mental mode. The present study examines the putative benefits of MT in University students for whom mind wandering can interfere with learning and academic success. We tested the hypothesis that short-form MT (7 h over 7 weeks) contextualized for the challenges and concerns of University students may reduce mind wandering and improve working memory. Performance on the sustained attention to response task (SART) and two working memory tasks (operation span, delayed-recognition with distracters) was indexed in participants assigned to a waitlist control group or the MT course. Results demonstrated MT-related benefits in SART performance. Relative to the control group, MT participants had higher task accuracy and self-reported being more “on-task” after the 7-week training period. MT did not significantly benefit the operation span task or accuracy on the delayed-recognition task. Together these results suggest that while short-form MT did not bolster working memory task performance, it may help curb mind wandering and should, therefore, be further investigated for its use in academic contexts.

*“The faculty of voluntarily bringing back a wandering attention, over and over again, is the very root of judgment, character, and will…An education which should improve this faculty would be the education par excellence. But it is easier to define this ideal than to give practical directions for bringing it about*.”

William James (1890)

## INTRODUCTION

Early in the history of modern psychology, James conveyed the insight that it is important to tame a wandering mind. Mind wandering is defined as task unrelated thought that occurs when there is a shift away from external stimuli and representations associated with ongoing activities and goals (Barron et al., 2011). It is linked to diminished cognitive performance (Smallwood and Schooler, 2006; McVay and Kane, 2009, 2012b; Kam and Handy, 2013) and corresponding disturbances in mood (Killingsworth and Gilbert, 2010). Mind wandering can be particularly problematic in academic contexts where success requires sustained attention to course content, as students must integrate information from external sources (e.g., from a lecture, text book, or class discussion) with ongoing internal representations and reactions that may or may not be related to academic learning (i.e., thoughts, memories, and emotions; Smallwood et al., 2007a). For example, if students are thinking about interactions they had just prior to entering a lecture (e.g., a difficult conversation with a friend) rather than the concepts the lecturer is explaining, they will likely fail to form robust and accurate internal representations of course concepts. Indeed, mind wandering has been demonstrated to impair memory for lecture material (Lindquist and McLean, 2011; Farley et al., 2013). Accordingly, providing an education *par excellence* may require training students in strategies to help them reduce mind wandering in the service of learning.

In addition to the learning challenges associated with off-task thinking, mood disturbances have been shown to negatively impact learning (Brand et al., 2007). Moreover, recent studies suggest that mind wandering and mood disturbances are also interrelated. Increases in negative mood causally follow episodes of mind wandering (Killingsworth and Gilbert, 2010) and negative mood inductions increase the incidence of mind wandering (Smallwood et al., 2009). Therefore, when developing strategies for improving classroom learning, it may be especially effective to target both attention and mood. One strategy used to promote learning in the University context involved offering mindfulness training (MT) to students (Ramsburg and Youmans, 2013). Students from an introductory psychology course were offered a brief mindfulness meditation session or a period of rest followed by a course lecture. After the lecture, students took a quiz assessing learning and retention of the material. Students in the mindfulness group outperformed students in the rest group, suggesting that engaging in a brief mindfulness exercise may bolster learning. Indeed, there is corroborating evidence that MT improves mood and attention (Jha et al., 2007; Baer, 2009) as well as decreases self-reported mind wandering (Mrazek et al., 2013).

Mindfulness is a mental mode characterized by attention to present moment experience without conceptual elaboration or reactivity. MT programs offer practices and didactic discussions on how to stabilize and focus attention on one’s present moment experience, as opposed to ruminating about the past or worrying about the future. A common practice offered in MT courses is mindfulness of breathing. Here, the participant is instructed to focus on a selected sensation of breathing (e.g., coolness of air in the nostrils) and maintain attention on that selected object for the period of formal practice. If a participant notices that his or her attention has wandered to off-task thoughts, feelings, sensations, or other internal preoccupations, he or she is instructed to guide attention back to the target object (e.g., the breath). Therefore, this type of training includes explicit instructions to notice mind wandering and respond by redirecting selective attention.

Given the centrality of addressing mind wandering in MT practices, prior studies have investigated if there are neural correlates to suggest that long-term MT practice may reduce mind wandering. A study by Brewer et al. (2011) compared long-term meditation practitioners’ and meditation naïve controls’ neural activity in the default mode network, a network of brain regions implicated in mind wandering and self-related processing (Gusnard et al., 2001; Mason et al., 2007; Christoff et al., 2009). The results suggested that this network was relatively deactivated in the long-term practitioners relative to novices during a formal meditation practice period in the scanner. Long-term practitioners also subjectively reported less mind wandering during meditation practice relative to controls. While this is consistent with the prediction that MT may reduce mind wandering, neural activity patterns, and subjective experience sampling alone are insufficient to conclude that MT reduces mind wandering. In addition, since this was a cross sectional study, the possibility that long-term practitioners may intrinsically differ in their default mode functioning rather than having acquired changes through their engagement in MT cannot be ruled out.

A recent study by Mrazek et al. (2013) investigated MT in a randomized control design with an active comparison group, and asked whether MT administered to novices improves task performance and reduces reports of the subjective experience of mind wandering. They offered a short-form MT course and a nutrition course (2 weeks, 6 h of in-class time) to undergraduate college students. Performance on verbal Graduate Record Examination (GRE) subsections was higher and self-reported mind wandering was lower in the MT group following training, a pattern not reported in the nutrition group. In addition to MT’s benefits on the GRE, a real-world measure of academic mastery, task performance on the operation span task (Unsworth et al., 2005), a laboratory measure of working memory capacity, was assessed. Consistent with other reports showing that MT bolsters working memory (Chambers et al., 2008; Zeidan et al., 2010; van Vugt and Jha, 2011), only the MT group improved in their operation span scores at the end of the 2 weeks course. Additionally, retrospective mind wandering reports completed following the operation span task suggested that the MT group and not the nutrition group showed lower mind wandering during the working memory task after the intervention than before it. A mediation analysis demonstrated that self-reported mind wandering significantly mediated the effect of MT on operation span and GRE performance, driven by those who had higher mind wandering prior to training. Accordingly, the authors concluded that the increase in performance may be attributable to a reduction in mind wandering. This proposal is consistent with a growing literature suggesting that working memory and mind wandering are interrelated (McVay and Kane, 2009, 2012a,b; Levinson et al., 2012).

While prior studies of MT in academic settings suggest it is beneficial, no studies have examined the impact of MT on optimizing task performance and reducing mind wandering *over the course of the academic semester* in University students. There is growing evidence that students’ stress levels and dysphoria increase over the semester, especially as they approach exams (Oaten and Cheng, 2005; Trueba et al., 2013). Off-task thinking has been related to psychological distress (Smallwood et al., 2005, 2007b; Stawarczyk et al., 2012), as well as negative vs. neutral mood (Smallwood and O’Connor, 2011) and as such, it is possible that mind wandering may increase over the semester as well. Here, we ask if MT may help to curb mind wandering that may result from rising academic pressures over the semester.

In order to inform strategies on how to best integrate MT into the academic semester, we investigated the efficacy of a short-form MT program specifically designed for University students with contextualized course content and practical features that considered students’ ongoing commitments (e.g., low time demands, convenient course location, flexible scheduling). To determine whether this form of MT protects against mind wandering, participants were given the sustained attention to response task (SART: Robertson et al., 1997), which successfully elicits mind wandering by requiring behavioral responses in the context of a monotonous, repetitive task with very low target probability, and low demand.

The SART was selected because of its long history as a neuropsychological assessment tool to index mind wandering (Robertson et al., 1997); its well-established neural and behavioral correlates (Christoff et al., 2009), as well its known test-retest stability (Robertson et al., 1997). The version of the SART used herein imbedded experience-sampling probe questions to explore the real-time (as opposed to retrospective) subjective experience of mind wandering, in addition to objective task performance. *Intra-individual reaction time variability* in the SART was also used, given growing evidence that it indexes mind wandering (Mrazek et al., 2012; Bastian and Sackur, 2013; Seli et al., 2013; Zanesco et al., 2013). For example, a set of studies using the SART showed that increasing intra-individual reaction time variability predicted subsequent self-reports of mind wandering, both through probe questions and asking participants to press a key anytime they noticed an attentional lapse (Bastian and Sackur, 2013). Together these SART outcome variables provide a useful profile of mind wandering and sustained attention.

Earlier studies suggest that MT is efficacious at curbing mind wandering. MT has been associated with reduced self-reports of mind wandering in highly engaging tasks (GRE and operation span, Mrazek et al., 2013) as well as during periods of MT practice (Brewer et al., 2011); moreover, 8 min of mindful breathing reduced mind wandering indicators during the SART, a pattern not seen when participants completed 8 min of rest or reading (Mrazek et al., 2012). However, to our knowledge no prior multi-session cognitive training study has investigated if SART performance improves with MT. In addition, two measures of working memory, the operation span task and a delayed-recognition task (Jha and Kiyonaga, 2010), were included to determine if prior demonstrations of MT’s benefits on working memory could be replicated when the time-demands of the course were reduced to 7 h over 7 weeks. We asked whether MT reduces mind wandering and increases task performance in a student cohort over the course of the academic semester. Based on the extant literature suggesting that academic and exam stress may set the stage for increases in mind wandering, we predicted that relative to a no-intervention control group, students who received MT would report experiencing less mind wandering and have better task performance on the SART as well as the working memory tasks.

## MATERIALS AND METHODS

### PARTICIPANTS

**FIGURE 1:**
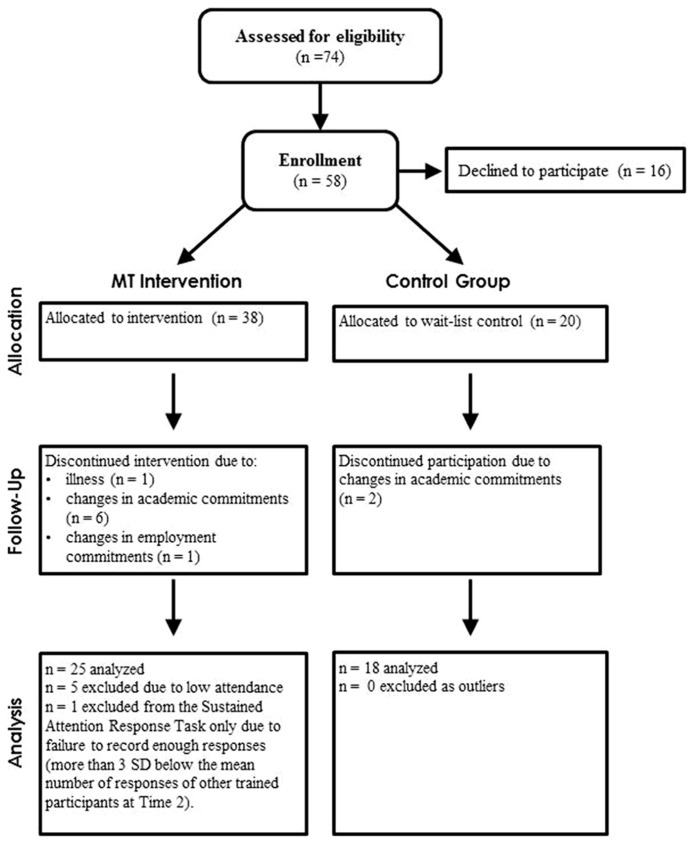
**Consolidated Standards of Reporting Trials (CONSORT; Schulz et al., 2010) flow diagram showing the number of participants in each of four phases, enrollment, treatment allocation, follow-up, and analysis**.

All participants were recruited at the beginning of the Fall 2012 academic semester at an orientation for psychology and neuroscience majors and through flyers on the University of Miami Campus. 58 (30 female) healthy University of Miami students (*M* = 18.20 years old, SD = 1.29) participated in an experiment testing the outcome of a mental training program over the academic semester and were assigned to either the MT group (*n* = 30) or a wait-list control group (*n* = 18). **Figure 1** presents a depiction of the flow of participants through group assignment, treatment allocation, and data analysis.

Assignment was quasi-random. All participants were drawn from the same volunteers and the only non-random factor determining group assignment was the student’s schedule. As the present study was meant to test the feasibility of a new intervention administered in partnership with a University-wide initiative, priority was given to placing at least 30 participants in the intervention group, and this led to unequal group sizes. The study was approved by the University of Miami Institutional Review Board, and informed consent was obtained prior to entry into this study.

### ASSESSMENT SESSION AND TASKS

**FIGURE 2:**
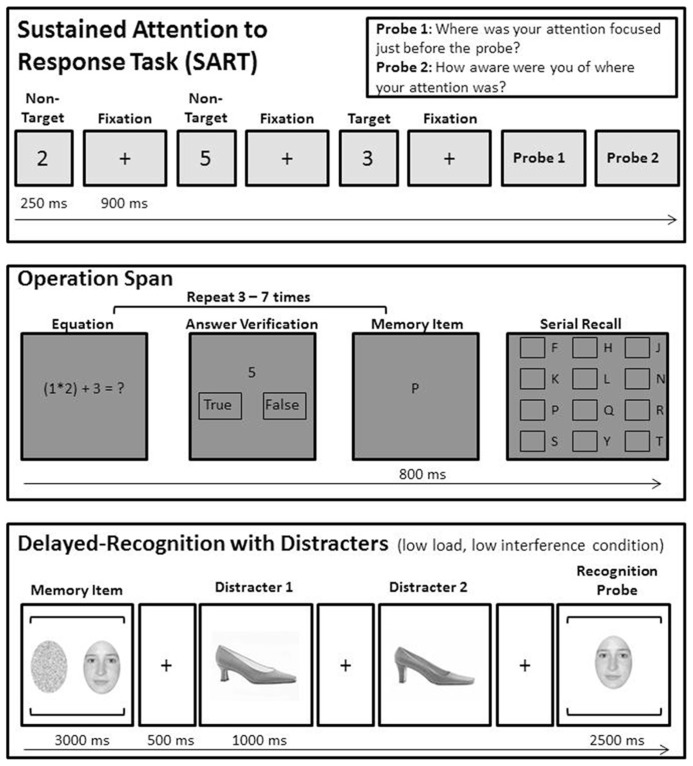
**Trial sequences and timing for the three computerized tasks that were administered prior to and following the 7 week training interval.** In the SART, participants viewed a string of single digit numbers and were instructed to key press in response to any digit except three (non-target) and to withhold response when a three was presented (target). During the task, self-report probes intermittently asked participants about attention and awareness. Participants responded on a Likert scale. In the operation span task, trials presented interleaved letters and math problems, and after each sequence, participants reported the letters in serial order. During the delayed recognition task participants were presented one or two memory items (faces or shoes), and after a series of two distracters, were asked whether a probe was an identical match to the memory item.

All participants completed two testing sessions, one before and another following the 7 week training interval. The first session corresponded with the first 2 weeks of the semester and the second session, which was in the period between midterms and final exams, occurred during a 2 weeks period that began 3 days after training ceased. Testing sessions involved a computerized battery of cognitive tasks administered by an experimenter in a group setting of up to six participants. Each participant was seated 57″ from a PC laptop display and stimuli were presented via E-Prime (Version 1.2; Psychology Software Tools, Pittsburgh, PA, USA). **Figure 2** visually depicts all three cognitive tasks.

#### SART

Sustained attention and self-reported mind wandering were measured through a modified version of SART (Robertson et al., 1997). Participants viewed a continuous array of single digits (0 through 9), and were told to withhold pressing the space bar to the number 3 (the target) and to respond to all other numbers (non-targets) by pressing the space bar. They were asked to respond as quickly as they could without sacrificing accuracy. Each trial included a digit displayed for 250 ms on a gray screen, followed by a fixation cross displayed for 900 ms. Participants could respond either during the stimulus display or during the intertrial interval. Targets were presented on 5% of trials. Trial order was pseudo-randomized so that target trials were always separated by at least one non-target trial.

On occasion, two probe questions were presented in succession. The first asked, “Where was your attention focused just before the probe?” Participants responded on a six-point Likert scale, where one represented “on-task” and six “off-task.” A second question asked, “How aware were you of where your attention was?” Participants responded on a similar scale, where one represented “aware” and six “unaware.” The probe questions were displayed until a response was made. There were 28 probes randomly dispersed throughout all 546 trials of the task. The task began with a 163 trial practice block that included 11 probes; the results from the practice block were not included in analyses.

SART yields a rich set of outcome variables. Task performance was quantified by examining mean overall accuracy, mean accuracy on target trials (i.e., trials showing the number, 3), mean accuracy on non-target trials (i.e., trials showing digits besides 3), and mean reaction time on correct non-target responses. Non-target errors are thought to suggest total disengagement from the task as they reflect a failure to button press in response to a stimulus (see Cheyne et al., 2009), while target errors are thought to reflect that the task is being performed in an automated rather than controlled fashion leading to a failure to withhold response to an infrequent target (Robertson et al., 1997). Overall accuracy provides a composite of both error types, and thus can be viewed as an indicator of multiple types of attentional failures during the SART.

Intra-individual reaction time variability in the SART was also used, given growing evidence that variability in response speed indexes mind wandering (Mrazek et al., 2012; Bastian and Sackur, 2013; Seli et al., 2013; Zanesco et al., 2013). Intra-individual reaction time variability was calculated as the standard deviation around an individual’s reaction times for correct non-target trials divided by this individual’s mean reaction time for correct non-target trials (i.e., for each participant: standard deviation RT/mean RT). The subjective experience of mind wandering was measured by calculating a participants’ mean response to each of the two types of embedded mind wandering questions.

#### Operation span

The automated operation span task was administered to assess working memory capacity (Unsworth et al., 2005). This complex span working memory task interleaves presentation of to-be-remembered letters, shown for 250 ms, with an unrelated decision task, a math problem to be verified. A trial included between three and seven cycles of letters and math problems, and at the end of each trial the letters were reported in serial order. The task includes 15 trials, 3 at each list length. The outcome variables are based on the number of items correctly recalled, and here we employ two scoring systems, an absolute score, where items are only correct if all items in the trial are correctly recalled in serial order, and the partial credit score, where credit is granted for items recalled in the correct serial position regardless of whether all items in a list are correctly recalled (Conway et al., 2005).

#### Delayed-recognition with distracters

A delayed-recognition working memory task measured short term retention of faces and shoes (Jha and Kiyonaga, 2010). This task includes parametric variation of two types of demand, memory load (high mnemonic load, low mnemonic load) and distracter interference (low distracter interference, high distracter interference). Each trial included the following sequence. (1) Presentation of a memory array with two items (high mnemonic load), or one item and a noise mask (low mnemonic load) appearing side by side. (2) A delay period where two task-irrelevant distracters were presented sequentially. Both distracter images within a trial were always of the same category (e.g., two faces or two shoes). In low interference trials the category of the memory items and distracters was mismatched while in high interference trials the category of the memory items and distracters was matched. (3) A test item was presented centrally for 2500 ms. On half of trials this was one of the memory items, and on half of trials it was a new item. After a series of 36 practice trials, subjects began the experiment, which comprised two experimental blocks of 30 trials each. This included 15 trials of each of the four conditions, low mnemonic load and low distracter interference, low mnemonic load and high distracter interference, high mnemonic load and low distracter interference, high mnemonic load and high distracter interference. The outcome variables for this task included accuracy and reaction time for each of the four conditions above, but the accuracy of item recognition is considered the dependent variable of interest since task instructions emphasized that participants should ensure accuracy in responding moreso than speed (Jha and Kiyonaga, 2010).

### TRAINING PROTOCOL

Course content was modeled after the core practices and concepts of mindfulness-based stress reduction (MBSR: Kabat-Zinn et al., 1992), highlighting topics relevant for the academic learning environment and including an introduction to core mindfulness concepts and to mindfulness practice. Content was developed and delivered by an expert mindfulness instructor. The 7 weeks training program included two components: instructor-led mindfulness based sessions and supervised practice sessions. Each week, participants attended a 20 min instructor-led session that was appended to an introductory seminar offered to psychology majors in their first semester. At these sessions, the instructor conveyed basic mindfulness concepts surrounding the following topics: defining mindfulness, cultivating focus and staying on task, acknowledging doubt and judgment, stress reduction, and integrating mindfulness into everyday life. Sessions also included mindfulness practice, and discussion of challenges arising during the academic semester. Each session closed with a 5–10 min practice session led by the instructor.

Participants then attended two separate 20 min individual practice sessions per week in the laboratory and listened to audio files on MP3 players with headphones in individual work stations. Sessions were supervised by a researcher, and these sessions were offered several times during the week so that students could choose a session that accommodated their schedules. During these sessions participants listened to attention-focusing audio files recorded by the instructor. Each week participants alternated between a mindful sitting practice and a body scan practice with the exception of the final week when participants selected their preferred practice.

Outside of the 20 min weekly course meeting and twice weekly 20 min proctored mindfulness practice sessions, no other requirements to practice mindfulness exercises were made. The course spanned 7 weeks over the Fall academic semester at a US University.

### DATA ANALYSIS

All significance tests were conducted from the perspective of null hypothesis significance testing (NHST) and were non-directional with alpha = 0.05. Analyses were designed to probe our question of interest: does MT promote measurable and significant cognitive benefits that are not seen in a group not receiving training? Toward this end, our central analyses were a series of repeated measures ANOVAs that tested the effect of time [time 1 (T1), time 2 (T2)], and group (training, control) for all outcome measures for the three tasks. Significant interactions of time and group were followed by paired sample and independent sample *t*-tests. To supplement the NHSTs, partial eta-squared were reported as an estimate of effect sizes for all ANOVA. In order to estimate the size of performance changes over time in the MT and control group, significant paired-samples *t*-tests were accompanied by Cohen’s *d* values.

Participants were excluded if they did not attend 75% of the total sessions, instructor-led and practice session, and this led to the exclusion of five participants from the training group. This threshold for inclusion was necessary as our research question was specific to the amount of time participants were engaged in mindfulness content. Excluded participants attended an average of 43% of sessions while included participants attended an average of 92% of sessions. Outlier identification was conducted by checking whether participants’ accuracy, reaction time, and number of responses were three standard deviations above or below the mean of the group (training, control) at a particular time point (T1, T2). This procedure resulted in removal of one participant from the SART who at T2 failed to respond to a sufficient number of non-target trials, suggesting that this participant failed to perform the task as instructed, and may have fallen asleep. All reaction time analyses included correct trials only, and removed reaction times under 200 ms.

## RESULTS

**Table 1** shows participant performance on all outcome measures at T1 and T2. At T1 there were no differences between groups in any of the outcome variables (all *p* ≥ 0.1).

**Table 1:** Cognitive performance prior to and following the 7 week training interval.

	Control			Training			Time × group interaction
	Group *n*	T1 *M* (SD)	T2 *M* (SD)	Group *n*	1T *M* (SD)	T2 *M* (SD)	*p*-Value
**SART**
Overall ACC	18	95.79% (2.59%)	90.61% (9.10%)	24	94.43% (3.45%)	95.59% (4.00%)	<0.01
Target ACC	18	52.23% (21.84%)	53.57% (19.24%)	24	44.51% (21.65%)	55.05% (22.50%)	0.16
Non-Target ACC	18	98.12% (1.95%)	92.54% (9.52%)	24	97.13% (2.86%)	97.75% (3.34%)	<0.01
RT	18	353.00 (47.77)	367.99 (42.62)	24	350.30 (58.80)	347.46 (52.63)	0.5
Intra-ind. RT Var.^*^	18	0.30 (0.09)	0.38 (0.15)	24	0.35 (.12)	0.32 (0.14)	<0.01
Probe 1^*^	18	2.11 (1.08)	2.61 (1.38)	24	1.85 (0.82)	1.78 (0.73)	0.02
Probe 2^*^	18	2.17 (1.05)	2.40 (1.13)	24	2.00 (0.95)	1.80 (1.03)	0.15
**Operation Span**
Partial Credit	18	46.67 (18.34)	46.72 (19.92)	25	45.76 (18.75)	53.68 (13.52)	0.11
Absolute Credit	18	61.94 (11.17)	63.33 (11.87)	25	60.40 (13.76)	66.08 (6.57)	0.23
**Delayed Recognition**
ACC	18	87.13% (6.73%)	86.64 % (9.26%)	25	90.39% (5.71%)	89.83% (5.49%)	0.99
RT (ms)	18	877.03 (155.46)	939.31 (193.40)	25	899.57 (178.48)	853.27 (142.39)	0.02

The group *n* (e.g., number of participants in each group) was the same at T1 and T2. SART, sustained attention response task; ACC, accuracy; RT, reaction time. Intra-ind. RT Var., intra-individual reaction time variability. ^*^Cases where a higher score is associated with higher degrees of inattention.

### SART

**FIGURE 3:**
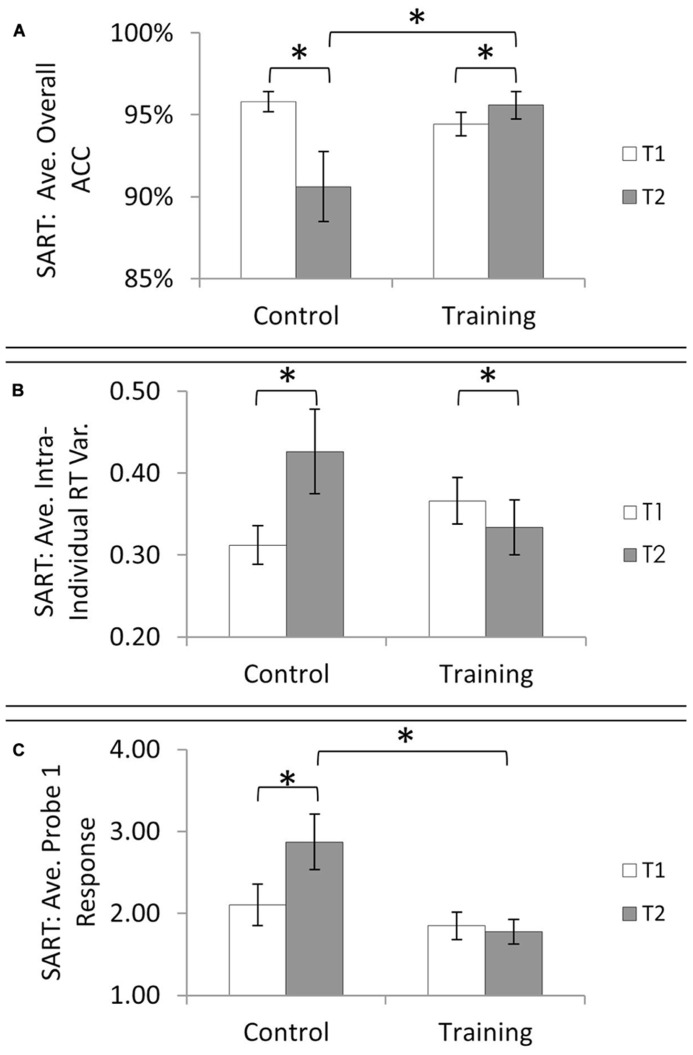
**Sustained Attention to Response Task (SART) performance overtime and between training groups on three outcome measures of the SART: (A) average overall accuracy, **(B)** average intra-individual reaction time variability, and **(C)** average response to Probe 1 which asked, “Where was your attention focused just before the probe?” A numerically higher probe response suggests more “off-task” thinking**. Asterisks denote that paired comparisons revealed significance at alpha of 0.05.

A series of ANOVAs examined time (T1, T2) by group (training, control), for all SART-related outcome variables. Overall accuracy revealed a significant decrease over time [*F*(1,40) = 5.16, *p* = 0.03, $\eta_{p}^{2}$ = 0.11], and interaction of time by group [*F*(1,40) = 12.82, *p* = 0.001, $\eta_{p}^{2}$ = 0.24], with no main effect of group [*F*(1,40) = 1.78, *p* = 0.19, $\eta_{p}^{2}$ = 0.04]. Paired comparisons indicated that the time by group interaction was driven by a performance increase over time in the training group [*t*(23) = 2.11, *p* = 0.046, *d* = 0.31 ) accompanied by a performance decrease over time in the control group [*t*(17) = 2.70, *p* = 0.02, *d* = 0.77; **Figure 3A**]. In addition, while the groups did not differ at T1 [*t*(40) = 1.40, *p* = 0.17], the training group had higher accuracy than the control group at T2 [*t*(40) = 2.40, *p* = 0.02].

Next, accuracy was subdivided into two error types, target accuracy (correctly withholding responses on trials with the number 3), and non-target accuracy (correctly pressing a button to indicate the presented digit is not a 3). For accuracy on target trials, there was no significant main effect of time [*F*(1,40) = 3.41, *p* = 0.07, $\eta_{p}^{2}$ = 0.08], group [*F*(1,40) = 0.28, *p* = 0.60, $\eta_{p}^{2}$ = 0.007] or interaction of time and group [*F*(1,40) = 2.04, *p* = 0.16, $\eta_{p}^{2}$ = 0.05]. For non-target trials there was significant decrease over time [*F*(1,40) = 7.73, *p* = 0.008, $\eta_{p}^{2}$ = 0.16], and interaction of time by group [*F*(1,40) = 12.07, *p* = 0.001, $\eta_{p}^{2}$ = 0.23], but no main effect of group [*F*(1,40) = 2.62, *p* = 0.11, $\eta_{p}^{2}$ = 0.06]. Performance did not change over time in the training group [*t*(23) = 1.50, *p* = 0.14], but decreased in the control group [*t*(17) = 2.80, *p* = 0.01, *d* = 0.81]. Moreover, while the two groups performed equivalently at T1 [*t*(40) = 1.3, *p* = 0.21], the MT group outperformed the control group at T2 [*t*(40) = 2.49, *p* = 0.02].

In the case of mean reaction time (ms), there was no significant main effect of time [*F*(1,40) = 1.93, *p* = 0.17, $\eta_{p}^{2}$ = 0.05], group [*F*(1,40) = 0.29, *p* = 0.60, $\eta_{p}^{2}$ = 0.007], or interaction of time and group [*F*(1,40) = 0.45, *p* = 0.51, $\eta_{p}^{2}$ = 0.01]. For intra-individual reaction time variability, a measure where higher amounts of variability are shown to index more mind wandering, there was no main effect of time [*F*(1,40) = 1.73, *p* = 0.20, $\eta_{p}^{2}$ = 0.04] or group [*F*(1,40) = 0.01, *p* = 0.91, $\eta_{p}^{2}$ = 0.00]. But, there was a significant time by group interaction [*F*(1,40) = 10.83, *p* = 0.002, $\eta_{p}^{2}$ = 0.213]. Paired comparisons within each group over time, revealed a decrease in intra-individual reaction time variability in the training group from T1 to T2 [*t*(23) = 2.20, *p* =0 .04, *d* = 0.23] and an increase in intra-individual reaction time variability in the control group from T1 to T2 [*t*(17) = 2.33, *p* = 0.03, *d* = 0.65; **Figure 3B**]. The training and control groups did not differ at T1 [*t*(40) = 1.40, *p* = 0.17] or at T2 [*t*(40) = 1.22, *p* = 0.23].

Analyses of the probe 1 responses revealed marginal effects of time [*F*(1,40) = 3.64, *p* = 0.06, $\eta_{p}^{2}$ = 0.08] and group [*F*(1,40) = 3.52, *p* = 0.07, $\eta_{p}^{2}$ = 0.08], and a significant interaction of time by group [*F*(1,40) = 6.50, *p* = 0.02, $\eta_{p}^{2}$ = 0.14]. Paired comparisons indicated that the time by group interaction was driven by no change over time in the training group [*t*(24) = 0.644, *p* = 0.52] and reporting of more off-task behavior in the control group at T2 vs. T1 [*t*(17) = 2.35, *p* = 0.03, *d* = 0.40; **Figure 3C**]. Moreover, while the groups did not differ at T1[*t*(40) = 0.87, *p* = 0.39], there were significantly more “off-task” responses in the control group vs. the training group at T2 [*t*(40) = 2.53, *p* = 0.02].

Probe 2 showed no main effect of time [*F*(1,40) = 0.05, *p* = 0.83, $\eta_{p}^{2}$ = 0.001] or group [*F*(1,40) = 1.88, *p* = 0.18, $\eta_{p}^{2}$ = 0.05] and no interaction of time and group [*F*(1,40) = 2.14, *p* = 0.15, $\eta_{p}^{2}$ = 0.05].

In summary, prior to training, there were no significant differences in any of the SART outcome variables (overall accuracy, target accuracy, non-target accuracy, Probe 1 and 2 responses, or intra-individual reaction time variability). More important, there was a significant group by time interaction for several of the performance and mind wandering variables (overall, non-target accuracy, Probe 1 responses, intra-individual reaction time variability). Independent *t*-tests between groups at T2 revealed that relative to the control group, MT led to improved overall and non-target accuracy on the SART and reduced self-reported mind-wandering as indexed by Probe 1, while group differences in intra-individual reaction time variability at T2 did not reach significance. To track changes over time within each group, paired samples *t*-tests found that overall accuracy increased and intra-individual reaction time variability significantly decreased from T1 to T2 in the MT group; whereas overall accuracy and non-target accuracy decreased, and mind-wandering probe responses and intra-individual reaction time variability increased in the control group.

### DELAYED-RECOGNITION WITH DISTRACTERS

A series of ANOVAs examined load (high, low) by interference (high, low) by time (T1, T2) by group (training, control) on accuracy and reaction time with two main aims, to determine whether performance varied with level of cognitive demand, and to characterize performance over time and across groups.

In the case of accuracy, performance varied with changes in cognitive load, confirming that difficulty varied by condition. Specifically, main effects of load and interference level were observed. Accuracy scores were higher on low load versus high load trials [*F*(1,41) = 66.55, *p* < 0.0005, $\eta_{p}^{2}$ = 0.62], and for trials with low versus high interference levels [*F*(1,41) = 101.69, *p* < 0.0005, $\eta_{p}^{2}$ = 0.71].There was no effect of time [*F*(1,41) = 0.33, *p* = 0.57, $\eta_{p}^{2}$ = 0.01], group [*F*(1,41) = 2.96, *p* = 0.093, $\eta_{p}^{2}$ = 0.07], or interaction of time and group [*F*(1,41) = 0.00, *p* = 0.99, $\eta_{p}^{2}$ = 00]. There were also no interactions of time and group with load and/or interference (*p* > 0.28).

For reaction time there was a main effect of load where participants were faster on low load trials [*F*(1,41) = 34.99, *p* < 0.0005, $\eta_{p}^{2}$ = 0.46], and a main effect of interference where they were faster on low interference trials [*F*(1,41) = 119.35, *p* < 0.0005, $\eta_{p}^{2}$ = 0.74]. There was no main effect of time [*F*(1,41) = 0.06, *p* = 0.82, $\eta_{p}^{2}$ = 0.00] or group [*F*(1,41) = 0.47, *p* = 0.50, $\eta_{p}^{2}$ = 0.01] but there was an interaction of time by group [*F*(1,41) = 6.01, *p* = 0.02, $\eta_{p}^{2}$ = 0.128]. Follow-up contrasts revealed that the training group was marginally faster from T1 to T2 [*t*(24) = 1.81, *p* = 0.08] while the control group’s reaction times did not differ between T1 and T2 [*t*(17) = 1.65, *p* = 0.12]. There were no other interactions (all *p* > 0.11).

### OPERATION SPAN

Analysis of time (T1, T2) by group (training, control) on the absolute score revealed no effect of time [*F*(1,41) = 2.77, *p* = 0.10, $\eta_{p}^{2}$ = 0.063], group [*F*(1,41) = 0.386, *p* = 0.538, $\eta_{p}^{2}$ = 0.009], or time by group interaction [*F*(1,41) = 2.70, *p* = 0.11, $\eta_{p}^{2}$ = 0.062]. Analysis of partial scores revealed overall improvement over time [*F* (1,41) = 4.01, *p* = 0.05, $\eta_{p}^{2}$ = 0.09], no effect of group [*F*(1,41) = 0.042, *p* = 0.839, $\eta_{p}^{2}$ = 0.001], and no interaction of time and group [*F*(1,41) = 1.50, *p* = 0.228, $\eta_{p}^{2}$ = 0.035].

## DISCUSSION

The current study investigated the impact of short-form MT in University student cohorts. Following a 7 week training interval during an academic semester at a US University, students engaging in MT showed greater sustained attention task performance and lower self-reported mind wandering during task completion than control students who received no training. The MT group demonstrated greater SART accuracy and lower intra-individual reaction time variability after the training period (T2) than before it (T1). In contrast, the control group demonstrated lower SART accuracy, greater self-reported mind wandering, and greater intra-individual reaction time variability at T2 than T1. Given that the SART is a stable task (Robertson et al., 1997), we do not attribute the observed patterns to task instability. Nor can the divergent SART patterns between groups be entirely attributable to a generalizable change in cognitive control processes, as neither of the two working memory tasks revealed any performance changes over time or group differences at either time point. Instead, we conjecture that MT may have strengthened a more specialized set of cognitive processes related to the control over mind wandering and sustained attention. Without such a specialized cognitive improvement, the control group more so than the MT group, may have been more vulnerable to psychological factors known to increase mind-wandering and known to change in student cohorts over the academic semester (i.e., greater intrusive thoughts, dysphoria, and reduced well-being; see Oaten and Cheng, 2005; Smallwood and O’Connor, 2011). Thus, the null effects on two measures of working memory, and the specificity of benefits to objective and subjective measures of mind-wandering with MT and costs without it, suggest that the short-form MT program herein, may have protected against a propensity for increased mind wandering over the academic semester.

The SART allows for measurement of two types of errors, non-target errors and target errors. Overall accuracy includes both of these error types, but as target trials are infrequent, non-target errors are more heavily weighted in this metric. Non-target errors are a failure to press a key in response to a very frequent event, and are thought to reflect complete inattention to and disengagement from the task (Cheyne et al., 2009). Target errors occur when a key is pressed incorrectly and are thought to reflect that the task is being performed in an automated manner where the infrequent targets go unnoticed (Robertson et al., 1997). Herein, students in the MT group showed improvement in an objective measure of mind wandering by achieving higher overall accuracy on the SART at T2 than T1, and higher overall accuracy than the control group at T2. Examination of only target accuracy did not reveal an effect of training (e.g., no significant time by group interaction). Meanwhile, examination of only non-target accuracy revealed higher non-target accuracy in the MT group than the control group at T2, achieved because the two groups were equivalent at T1, and there was a decline in the control group from T1 to T2, and no change in the MT group from T1 to T2. In short, the strongest evidence from the present study that MT benefits an objective measure of mind wandering is in the case of overall accuracy, a measure where both types of errors are considered together.

Trainees also showed a more consistent speed of responding (i.e., lower intra-individual reaction time variability) after training, a pattern found in two prior MT studies and interpreted as an indicator of more stable attention to the task (Lutz et al., 2009; Zanesco et al., 2013). Subjective reports of mind wandering during SART performance were indexed via probe questions presented throughout the SART itself. After the training period, the MT group had significantly less “off-task” reports compared to the control group, and they remained stable over time while the control group reported more “off-task” thinking from T1 to T2.

Control participants showed decreased SART accuracy, increased intra-individual reaction time variability, and increased reports of off-task thinking from T1 to T2, illustrating a consistent profile of degradation in sustained attention and increases in mind wandering in this student cohort over time. Notably, their decline in performance over time was only observed on the SART and not on the two working memory tasks. This task-specific degradation suggests to us that it may not represent an overall decrease in effort or motivation on the task battery for the control group. Instead, it may reveal selective degradation in sustained attention and a propensity toward mind wandering, which was successfully elicited in the context of the SART.

Consideration of effect sizes reveals that SART benefits in the training group over time corresponded to small effect sizes while decline in the control group over time corresponded to medium and large effect sizes. We argue that a small increase in the training group is made more compelling in the context of a larger decrease in those who did not train, and we cautiously suggest that this pattern supports an inter-relationship between resilience, sustained attention, and MT. The present results are sufficiently encouraging to warrant future studies to further explore how MT delivery might be optimized to promote larger benefits.

The current findings do not replicate the increases in working memory task performance reported in prior studies (Chambers et al., 2008; Zeidan et al., 2010; van Vugt and Jha, 2011; Mrazek et al., 2013); nor do they corroborate prior studies indicating a well-documented inter-relationship between working memory and mind wandering (see McVay and Kane, 2009; Levinson et al., 2012). Multiple accounts may explain why improvements in the SART were not accompanied by benefits to working memory performance. One such account involves the specific emphasis on mind wandering that is highlighted in the SART but not the other tasks. Self-report mind wandering probes were included in the SART, but not the WM tasks. The primary purpose of these probes is to gain insight into the subjective experience of mind wandering in real-time during the SART; however, these questions may have served an added purpose of alerting participants to their own wandering minds. During mindfulness practice in the MT course, the instructor would periodically remind participants to notice a wandering attention and then to return attention to the object of the exercise (e.g., the breath). During the SART, the mind wandering probes may have similarly reminded MT participants (and not untrained participants) to guide their attention back to the stimuli, which may have benefitted SART outcome variables. Notably, as no mind wandering probes were included in the working memory tasks, no such cues to shift attention back to the task were provided. Without these types of cues it is possible that mind wandering was not as robustly impacted during the working memory tasks, and accuracy on these tasks did not benefit from training.

Another difference between the SART and the two working memory tasks involves the perceptual load of the tasks. Mind wandering rates are shown to be lower in tasks with higher perceptual load than tasks with lower perceptual load (Antrobus et al., 1966; Forster and Lavie, 2009). The working memory tasks have a higher perceptual load than the SART (due to the requirements of encoding objects and equations compared to letters), and accordingly, the specific working memory tasks offered in the task battery herein may have promoted less mind wandering than the SART and may be a less sensitive measure of changes in mind wandering behavior.

Also, in the present study, MT may have benefited SART performance and not working memory performance due to similarities between SART and MT exercises. In both an MT exercise and the SART, one must attend to a repetitive string of stimuli, and notice and deter the mind’s tendency to wander away from the task. While the working memory tasks also require sustained attention and limiting of internal distraction, they additionally include external distracters (distracting images or math problems) and the requirement to encode, maintain, and retrieve memory items. Because this iteration of the SART does not contain these added components, it most closely approximates the set of processes required during the types of mindfulness exercises included in the present study. It follows that cognitive processes honed during mindfulness practice may be most likely to be applied to SART performance. Conversely, due to the added cognitive processes and task components in the working memory tasks, an individual may be less likely to show transferred benefits to working memory from MT. Put differently, SART can be considered “near transfer” from a short-form MT course; whereas, improvements in working memory could be considered “far transfer”.

A final explanation for the absence of working memory changes with the MT program herein is that the dosing of MT instruction time was insufficiently low to benefit working memory performance. Several of the studies reporting working memory benefits following MT included more intensive MT interventions with many more hours of course meetings and required practice time (Chambers et al., 2008; Jha et al., 2010; van Vugt and Jha, 2011). While Mrazek et al. (2013) also provided a short-form course, participants met with the instructor four times a week for a total of 6 h of instructor led content; whereas, in the present study, participants met with the instructor once a week for a total of 3 h and 20 min of instructor-led content. Thus, the short-form training provided in the present study may be been too low in its “dose” to instantiate such benefits.

The impetus for offering MT in the academic context came from the known deleterious effects of mind wandering on academic learning (Smallwood et al., 2007a; Farley et al., 2013), and prior reports of MT’s ability to reduce indicators of mind wandering (See Brewer et al., 2011; Mrazek et al., 2012). Yet, the challenge of offering MT in the University setting is that students have hectic schedules and may lack the motivation to invest their time in such training. In an attempt to limit attrition, while still promoting regular weekly engagement in MT practice, the course-related demands, timing, and structure were carefully tailored to best accommodate student participation. The MT course was offered to college students and integrated into their academic schedules by offering it for 20 min before or after a required freshman seminar. In addition, offering proctored training sessions in the laboratory eliminated the requirements for independent “at-home” practice and meant that students could engage in around 40 min of MT exercises a week, outside of the instructor led group session. Twenty five out of 30 students completed at least 75% of the sessions (and these 25 participants completed an average of 92% of the seven assigned hours of assigned MT). Thus, the program structure was able to successfully accommodate and achieve student participation in MT.

Nonetheless, limitations of the present study include the use of a wait-list control group rather than an active comparison group, and quasi-random group assignment which was tied to an individual’s personal schedule. We certainly agree that active controls and randomized designs are most prudent and remain the gold standards in the field. The present study represents our first attempt at partnering with an academic department and designing training as a companion to a required course, and a necessary next step is to compare this sort of training with an active control group matched on factors like instructor expertise and time commitment, and to randomly assign participants to training groups. Encouraging for the present results is the finding that Mrazek et al. (2013) administered the operation span task and self-report measures of mind wandering twice to an active comparison (nutrition training) and found neither an increase in operation span performance nor a decrease in self-reported mind wandering.

In the present study, there is evidence that MT successfully reduced mind wandering, as indexed during SART performance. Even so, it is important to note that we are not claiming that termination of all mind wandering is desirable or possible with MT. Indeed recently, there has been a call for a more a nuanced study of mind wandering where mind wandering may have both costs and benefits (Smallwood and Andrews-Hanna, 2013). Still, the results presented here highlight that when a task-at-hand is to be performed, and the task parameters make performance susceptible to mind wandering, MT may protect against it. Mind wandering was not assessed outside of the experimental context, so it is not clear whether students in the MT group experienced fewer episodes of mind wandering during everyday tasks such as reading or listening to a lecture. In addition, it is also not clear if MT altered the frequency of mind wandering that may be occurring without any deleterious effects on performing the task-at-hand. Emerging evidence suggests mind wandering that promotes constructive internal reflection may not be entirely pernicious (Immordino-Yang et al., 2012). Instead, it may play a crucial role in both autobiographical planning and creative problem solving (McMillan et al., 2013). Future studies will need to determine if MT alters the frequency of such episodes.

In sum, to our knowledge, the present study is the first to demonstrate that SART, an objective laboratory index of mind wandering, is sensitive to a multi-week MT program. Additional studies with larger numbers of participants and active comparison controls are required to confirm the MT curbs mind wandering and to further elucidate if and how mind wandering relates to psychological health and academic achievement in student populations. While preliminary, these results do suggest that MT may be a practical route by which to tame a wandering attention and its further consideration in the educational context is warranted.

## Conflict of Interest Statement

The authors declare that the research was conducted in the absence of any commercial or financial relationships that could be construed as a potential conflict of interest.
